# Evaluation of biomechanical gait parameters of patients with Cerebral Palsy at three different levels of gait assistance using the CPWalker

**DOI:** 10.1186/s12984-019-0485-0

**Published:** 2019-01-28

**Authors:** Luis Felipe Aycardi, Carlos Andrés Cifuentes, Marcela Múnera, Cristina Bayón, Oscar Ramírez, Sergio Lerma, Anselmo Frizera, Eduardo Rocon

**Affiliations:** 1 Department of Biomedical Engineering, Colombian School of Engineering Julio Garavito, Bogotá, Colombia; 2 Neural and Cognitive Engineering Group, CAR of the Spanish National Research Council (CSIC), Arganda del Rey, Madrid, 28500 Spain; 3 Department of Electrical Engineering, Federal University of Espirito Santo, ES, Vitória, 29075-910 Brazil; 4 Niño Jesús Hospital, Madrid, 28009 Spain

**Keywords:** Cerebral Palsy, CPWalker, Gait, Kinematics, Rehabilitation robotics

## Abstract

**Background:**

Cerebral Palsy (CP) is the most common cause of permanent serious physical disability in childhood. Although many platforms have been developed, so far there are still not precise guidelines for the rehabilitation of the population with CP. The CPWalker is a robotic platform for the rehabilitation of children with CP, through which they can start experiencing autonomous locomotion in the rehabilitation environment. It allows the possibility of free movement and includes physical and cognitive interfaces into the therapy. The main objective of this work is to evaluate the effects of the CPWalker-based rehabilitation intervention in children with CP by comparing different gait parameters before, during and after the use of the platform.

**Findings:**

The evaluation was divided in three stages where the gait parameters and symmetry indexes of eight subjects with CP were evaluated. In the first stage patients walked only with the help they receive normally in daily life. During the second stage they walked with the CPWalker and finally, in the third stage, they repeated their gait without the platform. In all stages they wore an inertial G-Sensor ^*Ⓡ*^ while walking through the hospital facilities. The results showed statistical significant differences in several spatio-temporal parameters, pelvic angles and general gait cycle parameters, with and without the use of the robotic device. For the eight patients: cadence, speed and stride length presented similar values when comparing before and after the therapy. However, they decreased during the intervention (both means and standard deviations). No significant differences were found in the symmetry indexes with the use of the platform. In spite of this, a reduction in the pelvic angles ranges and propulsion was observed.

**Conclusions:**

The effect of using the device was analyzed for spatio-temporal parameters, pelvic girdle angles and general gait cycle parameters. Among the eighteen initial parameters, seven presented a statistical significant difference when comparing stage 2 of the intervention with stages 1 and 3. Those changes showed the potential of the CPWalker to improve muscular strength and gait patterns of the patients with CP in the long term and to provide useful information for the design of the future generations of rehabilitation robotic devices.

## Background

Cerebral Palsy (CP) is a movement and posture development disorder, attributed to a defect or lesion in the brain that appears in infancy or early childhood [1]. CP is often associated with different physical and associative conditions as sensory deficits, cognitive impairments, communication and motor disabilities, behavior issues, seizure disorder, pain and secondary musculoskeletal problems [1]. CP is a condition that does not progress, meaning that the injury that caused the brain impairment will not get worse or change over time. However, some of the related conditions and co-mitigating factors can be treated. One of the most important and studied consequences is mobility impairment, mainly characterized by reduced speed and endurance or shortened step length during gait [2].

To improve motor function, several technological advancements have been introduced into the field of rehabilitation to complement conventional therapeutic interventions [3]. Among these techniques, robot-assisted gait training or other computer-assisted systems, have been implemented after technological adaptations in the pediatric field [4]. Preliminary results have supported the feasibility of these novel approaches in the clinical context [5, 6]. Clinical experience suggests that gait training in children with considerable cognitive deficits could be conducted even more effectively using robot-assisted therapy rather than conventional training [4].

The CPWalker [7] is a robotic platform designed to help children with CP to recover the gait function. The platform is composed of a smart hands-free walker and a lower limbs exoskeleton. It is based on the commercially available device NFWalker (Made For Movement, Norway) with some mechanical modifications to transform into an active rehabilitation platform. These modification resulted in the incorporation of four active systems to the device. Furthermore, it includes a Human-Robot interface (HRi) consisting of a set of sensors attached to the device. Its role is to establish the interaction with the user during the robot-based interventions, allowing the control of the device.

Even though the CPWalker platform is similar to others adopted before [5, 8], it intends to establish an integral rehabilitation model. This is achieved through: (i) allowing the possibility of free movement in a real environment; (ii) including Assist as Needed (AAN) strategies; and (iv) integrating the Central Nervous System (CNS) into the human–robot loop, which is known as a “Top-Down” approach [9].

## Methods

This paper presents the evaluation of the effects of a robotic-based rehabilitation intervention on the gait parameters of eight patients with CP. The robotic platform was implemented in an intensive rehabilitation intervention adjusted for each patient and defined by the rehabilitation team of the “Hospital Niño Jesús” (Madrid, Spain). To evaluate the clinical validation and short term effects of this robotic device, the G-Walk protocol of the inertial G-Sensor ^*Ⓡ*^ (BTS, Italy) was implemented along with the CPWalker platform in the experimental procedure.

### Population

A total of eight subjects with CP (specifically with spastic diplegia) or Acquired Brain Injury (ABI) were recruited for this study. Four participants were female and four were male. In order to analyze a possible relation between the level of autonomy and general gait cycle indexes, all the patients were classified in three categories according to their level of autonomy and independence during the gait cycle: patients who needed human support to walk, patients who walked with crutches and patients who could walk by his/her own means without aid(s) (Table 1).

**Table 1:** Patients information

No	Disease	Age	Weight	Gender	Category
1	Spastic CP	12	41.9 Kg	Male	With crutches
2	Spastic CP	12	38.5 Kg	Male	With crutches
3	Spastic CP	13	36.7 Kg	Female	Support needed
4	ABI	8	44.0 Kg	Female	Support needed
5	Spastic CP	16	49 Kg	Male	Support needed
6	Spastic CP	14	41 Kg	Male	Support needed
7	ABI	13	71 Kg	Female	No aid required
8	Spastic CP	13	41.5 Kg	Female	No aid required

The inclusion criteria were: a) aged between 6 - 15 years-old; b) maximum weight 75kg; c) able to signal pain, fear or discomfort. The exclusion criteria of this study were defined as: a) unhealed skin lesions in the back or lower limbs; b) aggressive behaviors; c) severe cognitive impairment. The clinical trial was carried out at the “Hospital Infantil Universitario Niño Jesús”, where the local ethical committee gave approval to the study and warranted its accordance with the Declaration of Helsinki. All patients were informed beforehand and signed a written informed consent (through their parents or legal guardian) to participate.

### Robotic platform and experimental set up

The CPwalker has incorporated four active systems to be considered an active rehabilitation platform: (i) a drive system of the platform; (ii) a partial body weight support (PBWS) system; (iii) an active system for the adaptation of hip height; and (iv) a system for controlling joint motion of the exoskeleton.

In this study, all of them were included actively, except for the exoskeleton’s joint control system that acted passively. On the other hand, from the set of sensors integrated in the original HRi [7] (an electroencephalographic (EEG) acquisition unit, inertial measurement units (IMUs) and a Laser Range Finder (LRF)), only the LRF was included in the intervention. As part of the drive system, this sensor was used to measure the user’s leg’s kinematics. The leg detection algorithm and control loop evaluated in this study have been presented in previous work [10]. In the legs kinematic module, the velocity estimation is obtained from the distances measured from the right and left legs by the LRF. Thus, the control strategy conducted, aimed to provide a natural movement according to the level of disability by controlling the velocity of the translation through the movement of the lower limbs, without restricting the gait at any moment.

**Fig. 1:**
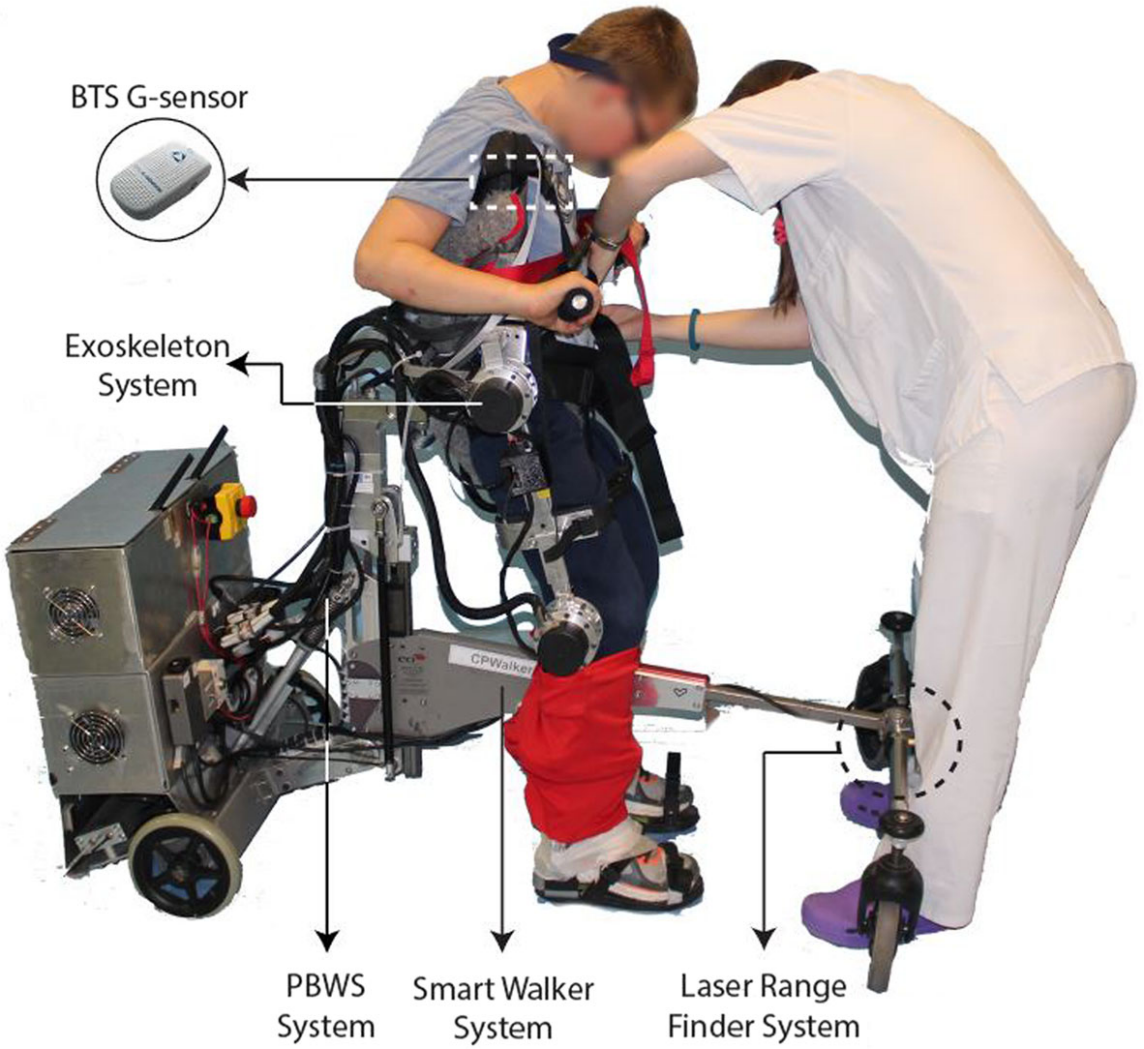
The CPWalker platform (exoskeleton, PBWS, smart walker and LRF systems) and the G-sensor ^*Ⓡ*^ for validation

The set up of the intervention was completed with the integration of the validated [11] wireless inertial sensing device (BTS G-Sensor ^*Ⓡ*^). The sensor is made up of 4 inertial platforms (Micro-Electro-Mechanical Systems, or MEMS) each of them composed by a triaxial accelerometer, a magnetic sensor, a triaxial gyroscope and a temperature sensor combined with advanced algorithms provided by the Sensor Fusion technology and of a GPS. It allows the acquisition and transmission of data to the PC through a Bluetooth connection and includes several analysis protocols. Specifically, the BTS G-Walk ^*Ⓡ*^ protocol allows a rapid and precise analysis of the gait and represents an ideal solution to evaluate pathologies that adversely affect this movement. The protocol provides (i) Spatio-temporal parameters (global and differentiated for right and left sides); (ii) General kinematic parameters (global and differentiated for right and left sides); (iii) Symmetry index, propulsion index; and (iv) Pelvic angles. The set up is depicted in Fig. 1.

### Rehabilitation intervention

**Fig. 2:**
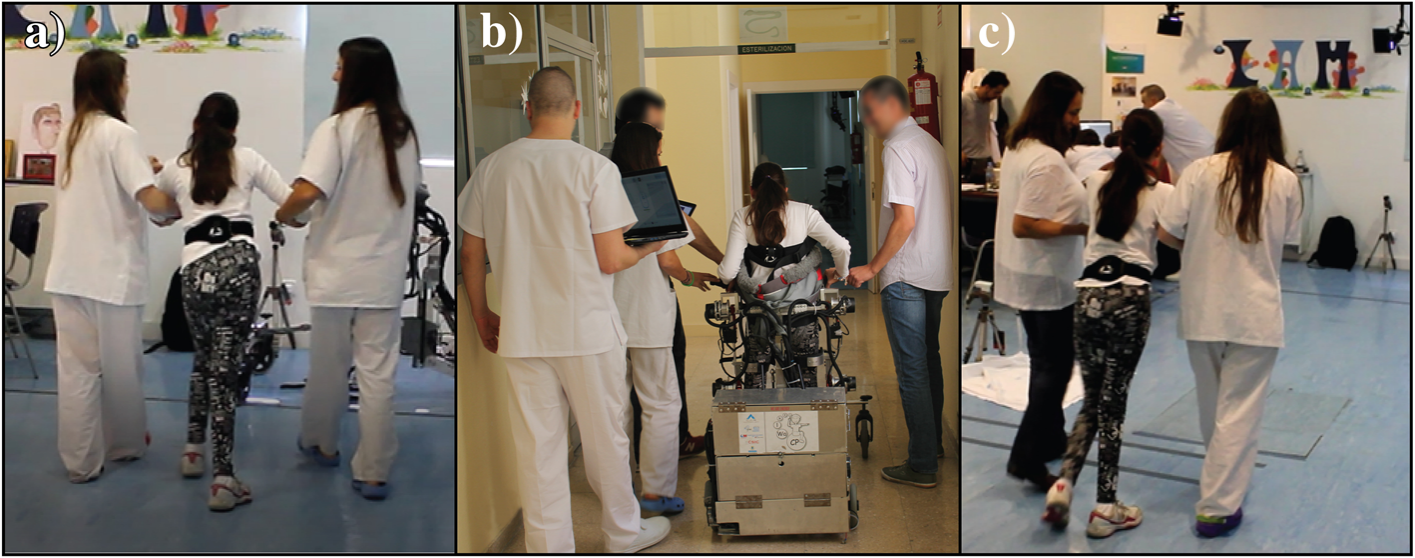
Experimental stages proposed. **a**) First stage, with patients helped according to the classification provided in Table 1**b**) Second stage, with patients using the CPWalker platform and **c**) Third stage, with the same conditions as the first stage

The procedure was divided in three stages, where the patient’s gait parameters were evaluated (Fig. 2). First, the patients walked in straight line for 1 to 2 min in a hospital room with the sensor located around the waist, without the robotic platform and with only the help according to the classification provided in Table 1 (Fig. 2a.). Subsequently, the patients walked with the robotic device (and the G-sensor) through straight routes in the hospital facilities for approximately 10 to 15 min. As previously stated, in this stage no control actions were implemented with the exoskeleton. On the contrary, the smart walker did actuate according to what the control loop had suggested [10] (Fig. 2b.). It is important to mention that the platform was adjusted to the hip height of each user, so that the PBWS system supported both the patient’s and exoskeleton’s weight, without lifting the patients to any degree. Thereby, the intervention was performed entirely overground, discarding any effect coming from the actuators. Finally, after using the CPWalker, patients repeated the first stage (Fig. 2c).

The three stages were conducted in sequence, in a single day per patient. The intervals between stages were conditioned to the instrumentation and adjustment times of the platform to each patient, which were roughly 12 to 20 min. The routes of each stage implicated having to turn in fixed places and continue walking in straight-line. For example, the second stage took place in the communal hallway of the hospital (aprox. 200 mts) with one 180°-turn included. Nonetheless, all the turns were discarded in the gait measurement and only the parameters recorded during the straight-line walking were considered.

Information recorded with the G-Walk ^*Ⓡ*^ protocol (Table 3) was gathered, compared and analyzed in the study to accurately describe the possible incidence of the platform in the gait biomechanics. The propulsion is defined as the difference in the anterior/posterior acceleration of the body barycenter (during the single support phase of the right and left side’s gait cycle), between the toe off to the heel strike events. The pelvic angles refer to a gyration each on a specific plane: the tilt on the sagittal plane, obliquity on the coronal plane, and the rotation on the transversal plane. The symmetry indexes presented (in the general gait cycle and pelvic angles) are the percentage of symmetry between either the curve of acceleration or each pelvic angle of the right and left gait cycle. As the indexes approach 100, the more symmetry there is along the way.

**Table 2:** Speed in the second stage of the experiment

Patient	Category	Avg. ± Std. Deviation
1	With crutches	0.265 ± 0.01
2	With crutches	0.203 ± 0.027
3	Support needed	0.361 ± 0.097
4	Support needed	0.211 ± 0.054
5	Support needed	0.202 ± 0.056
6	Support needed	0.111 ± 0.040
7	No aid required	0.315 ± 0.018
8	No aid required	0.294 ± 0.016

**Table 3:** Gait parameters evaluated in the three stages with the G-sensor ^*Ⓡ*^. Statistical results of the comparisons established. L (Left) and R (Right) sides

Gait parameters in G-Walk protocol			Statistical results of intervention						
Category	Parameter	Units	*	Avg. ± SD	Avg. ± SD	Avg. ± SD	p-value	p-value	p-value
				Stage 1 (S1)	Stage 2 (S2)	Stage 3 (S3)	S1/S3	S1/S2	S2/S3
Spatio-temporal parameters	Cadence	step/min	✓	102.5 ± 41.4	42.7 ± 11.2	89.5 ± 31.9	0.203	0.015*	0.015*
	Speed	m/s	✓	1.21 ± 0.47	0.32 ± 0.10	1.06 ± 0.33	0.148	0.007*	0.015*
	Stride length	m	✓	1.46 ± 0.24	0.94 ± 0.29	1.46 ± 0.22	0.984	0.015*	0.015*
	% Stride length	%height	-	-	-	-	-	-	-
	Gait cycle dur. (L/R)	s	-	-	-	-	-	-	-
	Step length (L/R)	%stride	-	-	-	-	-	-	-
	Stance phase (L/R)	%cycle	-	-	-	-	-	-	-
	Swing phase (L/R)	%cycle	-	-	-	-	-	-	-
	Single support (L/R)	%cycle	-	-	-	-	-	-	-
	Double support (L/R)	%cycle	-	-	-	-	-	-	-
Pelvic angles	Tilt sym. index	%	-	-	-	-	-	-	-
	Obliquity sym. index	%	-	-	-	-	-	-	-
	Rotation sym. index	%	-	-	-	-	-	-	-
	Right tilt range	deg (°)	✓	3.54 ± 2.38	0.88 ± 0.74	3.37 ± 1.81	0.781	0.007*	0.023*
	Left tilt range	deg (°)	✓	3.78 ± 2.46	0.93 ± 0.77	3.45 ± 1.92	0.656	0.023*	0.039*
	Right obliquity range	deg (°)	✓	2.93 ± 2.24	0.41 ± 0.37	3.43 ± 2.32	0.156	0.015*	0.015*
	Left obliquity range	deg (°)	✓	3.71 ± 2.04	0.46 ± 0.51	3.24 ± 2.36	0.843	0.015*	0.015*
	Right rotation range	deg (°)	✓	11.1 ± 6.41	2.38 ± 1.33	10.3 ± 4.65	0.734	0.015*	0.007*
	Left rotation range	deg (°)	✓	9.96 ± 5.81	2.16 ± 1.44	10.2 ± 4.13	0.496	0.015*	0.007*
General gait cycle parameters	General sym. index	%	-	-	-	-	-	-	-
	Right propulsion	m/s^2^	✓	8.41 ± 2.79	4.37 ± 2.33	8.42 ± 2.30	0.941	0.023*	0.023*
	Left propulsion	m/s^2^	✓	7.47 ± 2.99	3.91 ± 1.75	8.18 ± 2.19	0.945	0.031*	0.015*

^*^Significant difference shown when comparing the stages of the study

Statistical analysis was required to differentiate those parameters that presented a significant change in at least 2 stages of the experiment. A Kolmogorov-Smirnov test was realized for each group of variables (each parameter in stages 1, 2 and 3) and no normal distribution in either was obtained, conditioning the data to a median difference test to perform the comparison, a Wilcoxon signed-rank test (*α*= 5%).

## Results and discussion

This section presents, first, the comparison of the speed behavior provided by the smart walker’s control strategy and actual patient performance during the second stage of the intervention; and second, the gait parameter’s measures taken for the eight patients on the three stages of the experiment.

**Fig. 3:**
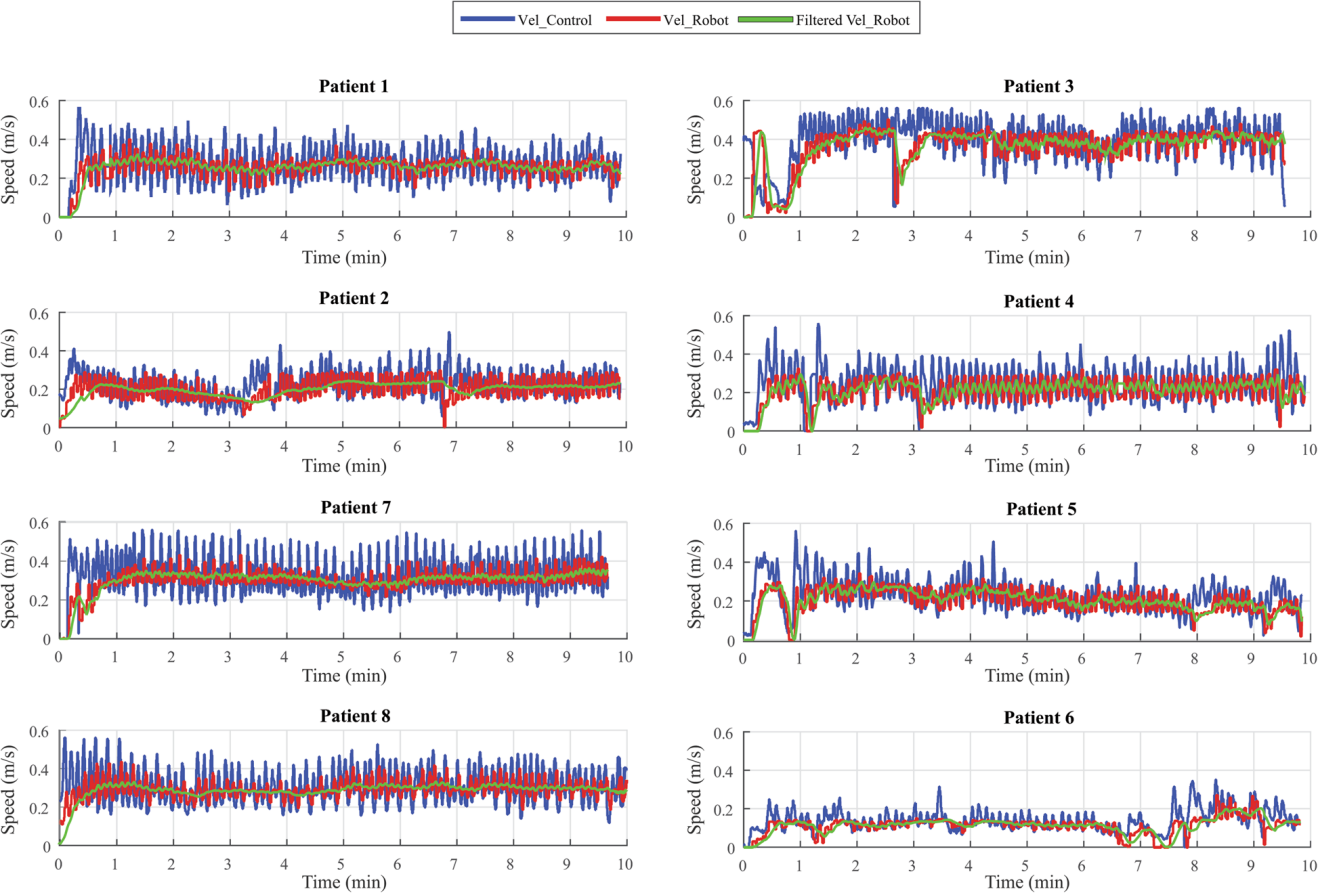
Speed behavior in the second stage of the intervention for patients with crutches (Patient 1 and Patient 2), patients with human support during gait (Patient 3, Patient 4, Patient 5 and Patient 6) and patients without any support needed (Patient 7 and Patient 8). The Blue line corresponds to the velocity proposed by the LRF control loop, the Red line corresponds to the velocity executed by the CPWalker and the Green line corresponds to a filtered robot velocity

Figure 3 presents the speed measurement during the experiment. Since this parameter was monitored in the second stage of the procedure, where not all of the patients walked the exact same time, the first 10 min of each patient’s exercise are shown to analyze the start up conditions and average values. The Vel_Control signal is the speed proposed by the LRF after going through the leg kinematic control loop; the Vel_Robot signal corresponds to the speed executed by the CPWalker; and the Filtered Vel_Robot signal is the result of a post-processing filter (moving average filter) of the Vel_Robot signal. As it can be seen, the Vel_Robot signal follows the Vel_Control signal correctly. This shows how the platform adapts to each patients gait and provides the AAN during the intervention. Average values and standard deviations were found for each performance from the Filtered Vel_Robot after the signal had stabilized (Table 2).

As it can be observed, the speed signal for patients with human aid during gait (showed in the right column of Fig. 3) presented some false starts and an overall instability of the signal, also evidenced by an increase of the standard deviation of this signal. This instability could be due to a lack of control in those patients reflected in the independence level they present during gait. The effect of the CPWalker platform, stabilizing the speed could be an important factor in the rehabilitation as the patients could learn a more steady gait pattern, usually modified by the selective motor control suffered by those patients [12].

In addition, in the left column of Fig. 3 the speed signal for patients with crutches and patients who walk without any support showed a value with lower standard deviations and with less time of stabilization. Specifically, among these two types of patients, the ones that did not require any aid, presented a higher speed average over patients with crutches (with similar standard deviation). This fact demonstrates the ability of these patients to perform a more independent, natural and continuous gait; and are inklings of a more convenient performance of the control loop of the walker, related to a better posture of those patients during the experiment with the platform. The effects are similar to the results found by other Robotic-Assisted Gait Training interventions (RAGT) [13].

On the other hand, the statistical analysis on the parameters measured in the three stages of the intervention with CPWalker showed that some of those parameters were not affected by the use of the robotic platform. In the spatio-temporal parameters, the platform did not modify parameters regarding the gait phases including the stance, swing, double support and single support durations. This could be due to the configuration of the platform with a passive exoskeleton and an active walker, where the mechanical structure can modify the posture of patient and restrict the speed but it does not modify the gait pattern itself. This parameter could be improved by the use of the exoskeleton on its active configuration in the future.

Concerning the pelvic angles and the general gait cycle parameters, the statistical analysis did not show differences for the symmetry indexes with the use of the platform, despite the differences in the ranges discussed later in this paper. This statistical finding could mean that even when the patient presents a smaller range of movement in the pelvic angles thanks to the restriction of the device, this does not disallow the gait asymmetry presented in those patients. This could be an improvement opportunity for the device design. Those findings are in agreement with a previous study between different type of deficits (diplegia/hemiplegia) where it was shown that the range of motion of the thorax and spine exhibited more significant differences between groups than the mean positions [14].

Finally, seven parameters showed a significant difference between the different stages of the experimental protocol, and were selected to analyze the effects of the platform. Those parameters were three spatio-temporal parameters, three pelvic angles parameters (each for both left and right sides) and one general gait cycle parameter (also for both sides), shown in Table 3. Such parameters presented a statistical significance difference using a Wilcoxon test (alpha = 0.05).

**Fig. 4:**
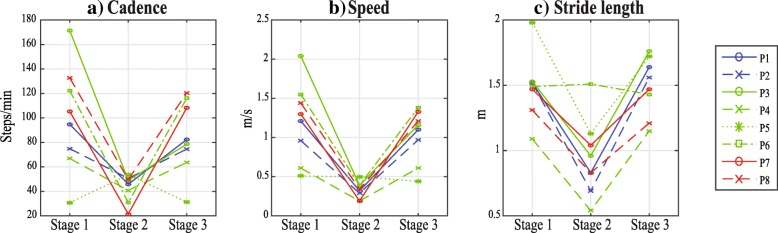
Spatio-temporal parameters. **a**) Cadence, **b**) Speed and **c**) Stride length measured at the 3 different stages of the intervention for the 8 patients

The spatio-temporal parameters that showed a significant difference between the experimental stages are cadence, speed and stride length (Fig. 4). For almost all patients (seven out of eight) those parameters presented a decrement in the second stage of the experiment. For the eight patients, cadence, speed and stride length presented similar values when comparing the first and third stage of the trial, but each stage presented a relative high standard deviation. However, in the second stage, they decreased and ended up with a lower standard deviation. This change could be explained taking into account the control strategy of the robot [10] which sets a maximum speed for all the patients in order to assure safety in the use of the robot, as those patients present in general, a reduced speed and a shortened step length [2]. Also, the structure of the exoskeleton itself played a role restraining the movement, even though the design of the PBWS considerably lessens this effect. Considering that stride length and cadence are directly related to gait speed [2], both parameters also decreased with the reduction of speed. However, the patients did not present a significant difference in those parameters when comparing stages 1 and 3 (Table 3), which could indicate that these parameters are not modified in the short term.

**Fig. 5:**
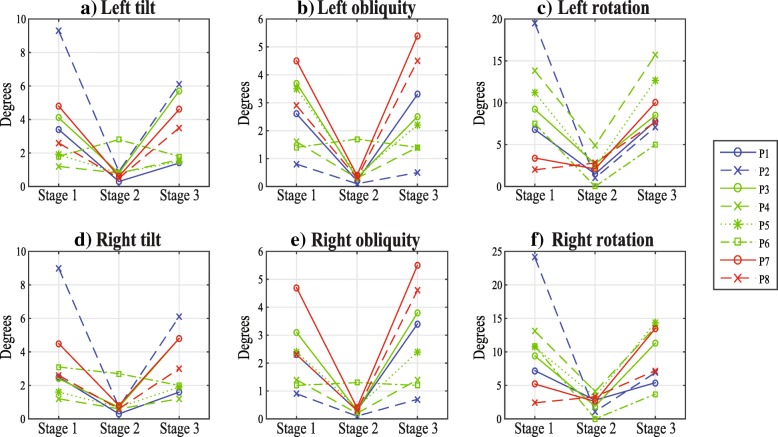
Pelvic angles. **a**) Left tilt, **b**) Left obliquity, **c**) Left rotation, **d**) Right tilt, **e**) Right obliquity and **f**) Right rotation measured at the 3 different stages of the intervention for the 8 patients

For the pelvic angles (Fig. 5), tilt, obliquity and rotation for both sides presented a difference during the use of the CPWalker but not after its use (Table 3). While using the CPWalker, patients presented a decrement on the pelvic angles, and, equivalently to the spatio-temporal parameters, they presented high deviation values in the first and third stages but they decreased during the second stage. This behavior could be due to the physical restriction of the device in the trunk movement. This restriction could also be an advantage in the rehabilitation process as it could reduce the fatigue in the lumbar muscles of the patient and it could improve the gait pattern, however further studies are required to assess this hypothesis.

**Fig. 6:**
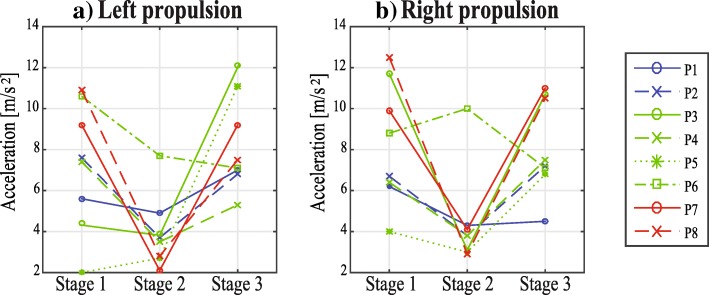
**a**) Left propulsion and **b**) Right propulsion measured at the 3 different stages of the intervention for the 8 patients

Finally, concerning the propulsion for the right and left side (Fig. 6), a difference was found stage 1/stage 2 and stage 2/stage 3 (Table 3). The decrease observed in the change of the acceleration during the single support phase could be due to the increase on the force necessary for the patient to walk as it has the weight of the device restricting that movement. This could be viewed as a disadvantage of the CPWalker, nevertheless, this may be corrected in the future with the use of active joint in the exoskeleton.

A Holm-Bonferroni post-hoc test was applied to the three comparison groups for each parameter. Only the parameters Left tilt range, Right rotation range and Right propulsion showed a probability of at least one Type I error in a set (family) of comparisons. For the other parameters this test showed results in coherence with the Wilconxon test.

## Conclusions

In this paper, a robotic-based rehabilitation intervention is set up for children with cerebral palsy. Three different levels of autonomy and independence during the gait cycle were evaluated: i) patients who needed human support to walk, ii) patients who walk with crutches, and iii) patients who can walk by his/her own means without aid(s). The effect of the use of the device was analyzed for spatio-temporal parameters, pelvic girdle angles and general gait cycle parameters. Seven of eighteen analyzed parameters presented a statistical significant difference during the use of the CPWalker compared to stages 1 and 3 of the intervention. Those changes were principally due to the physical restriction of the platform and they are only seen in the short term. However, the changes here analyzed are the first glimpse of the potential this platform has to improve muscular strength and gait patterns of patients with CP and similar motor disorders (including other types of ABI) in the long term. These results help to show some light on how robotic devices can rehabilitate the gait of children with CP and generate important information to help to improve the design of future version of these platforms. Even though outcomes are promising, it is clear that the reduced time of intervention is a restriction of this research. Future work will be focused on performing a clinical evaluation with an increased number of sessions, so that the results can be integrated to the current state of art of robotics gait rehabilitation. These studies are necessary to determine if the effects will have a lasting impact for children with CP.

## Abbreviations

AAN: Assist as Needed
ABI: Acquired brain injury
CNS: Central nervous system
CP: Cerebral palsy
HRi: Human-Robot interface
LRF: Laser Range Finder
PBWS: Partial body weight support
RAGT: Robotic-Assisted Gait Training
